# Protection of a defensive symbiont does not constrain the composition of the multifunctional hydrocarbon profile in digger wasps

**DOI:** 10.1098/rsbl.2023.0301

**Published:** 2023-11-01

**Authors:** Chantal Selina Ingham, Tobias Engl, Martin Kaltenpoth

**Affiliations:** ^1^ Department of Evolutionary Ecology, Institute of Organismic and Molecular Evolution, Johannes Gutenberg-University Mainz, Hanns-Dieter-Hüsch-Weg 15, 55128 Mainz, Germany; ^2^ Department of Insect Symbiosis, Max-Planck-Institute for Chemical Ecology, Hans-Knöll-Str. 8, 07745 Jena, Germany

**Keywords:** hydrocarbon, nitric oxide, symbiosis, *Streptomyces*, mutualism, *Philanthus*

## Abstract

Hydrocarbons (HCs) fulfil indispensable functions in insects, protecting against desiccation and serving chemical communication. However, the link between composition and function, and the selection pressures shaping HC profiles remain poorly understood. Beewolf digger wasps (Hymenoptera: Crabronidae) use an antennal gland secretion rich in linear unsaturated HCs to form a hydrophobic barrier around their defensive bacterial symbiont, protecting it from brood cell fumigation by toxic egg-produced nitric oxide (NO). Virtually identical HC compositions mediate desiccation protection and prey preservation from moulding in underground beewolf brood cells. It is unknown whether this composition presents an optimized adaptation to all functions, or a compromise due to conflicting selection pressures. Here, we reconstitute the NO barrier with single and binary combinations of synthetic linear saturated and unsaturated HCs, corresponding to HCs found in beewolves. The results show that pure alkanes as well as 3 : 1 mixtures of alkanes and alkenes resembling the composition of beewolf HCs form efficient protective barriers against NO, indicating that protection can be achieved by different mixtures of HCs. Since *in vitro* assays with symbiont cultures from different beewolf hosts indicate widespread NO sensitivity, HC-mediated protection from NO is likely important across Philanthini wasps. We conclude that HC-mediated protection of the symbiont from NO does not exert a conflicting selection pressure on the multifunctional HC profile of beewolves.

## 1. Introduction

Cuticular hydrocarbons (CHCs) protect insects from desiccation, and have often evolved secondary functions, e.g. intra- and interspecific communication in social and solitary insects [1–3]. Constituents of insect CHC profiles include n-alkanes, methyl-branched alkanes, alkenes and alkadienes of different chain lengths [2], forming complex compositions with up to more than 100 different components [1]. The chemical composition influences the function of the profile [3]. One profile frequently fulfills multiple functions, e.g. cuticle lubrication [4], prey protection from pathogens [5,6] and enhancing tarsal adhesion [7], posing potentially conflicting requirements on the CHC composition [3]. However, due to its complexity, it remains poorly understood how composition influences function, and how natural selection shapes composition considering the functional constraints [3].

An intriguing multifunctional hydrocarbon (HC) profile has recently been reported for a group of solitary digger wasps [6,8]. These beewolves (Hymenoptera: Crabronidae, *Philanthus triangulum*) construct brood cells in sandy soil and mass-provision their offspring with paralysed honeybee workers (*Apis mellifera*) [9–11]. Females embalm the bees in an alkene-rich HC secretion from their postpharyngeal gland (PPG), which prevents water condensation and thereby reduces fungal infestation [5,6,12]. Two further adaptations to protect the offspring have evolved in beewolves: first, females secrete defensive ‘*Streptomyces philanthi*’ symbionts from specialized antennal gland reservoirs into the brood cell [13,14]. After integration into the larval cocoon [13], symbiont-produced antibiotics provide efficient long-term protection against opportunistic microbes [13,15]. Second, the beewolf egg sanitizes the brood cell with toxic nitric oxide (NO) [16], preventing pathogen growth without harming the symbionts present in the brood cell during NO fumigation [8]. Recently, the HCs in the antennal gland secretion (AGS) [17] were found to form a hydrophobic barrier against NO around ‘*S. philanthi*’ [8].

The nearly identical HC profiles of the cuticle, the PPG and the AGS of *P. triangulum* are characterized by an approximately 3 : 1 alkene–alkane ratio, with compounds ranging from C21 to C31 in chain length [17–20]. Tricosane (C23) represents the most abundant alkane [17–20], and either pentacosene (C25 : 1) or heptacosene (C27 : 1) constitute the most abundant alkene [17,19,20]. Together with tricosane, C25 : 1 or C27 : 1 account for 51–92% of HCs [17–20]. Thus, desiccation protection, prey preservation and symbiont protection are realized by virtually the same profile (electronic supplementary material, table S1, figure S1) [17–20]. However, it remains unclear whether the alkene-rich HC profile presents an adaptation to all three functions, or a compromise arising from conflicting selection pressures. Here we tested single and binary mixtures of synthetic linear saturated and unsaturated HCs, corresponding to those found in beewolves, for their effectiveness in blocking NO. We demonstrate that a range of individual HCs, and mixtures of alkenes and alkanes resembling beewolf HC extracts, are effective NO barriers *in vitro*. Additionally, we show that symbiont strains from multiple different host species are susceptible to NO *in vitro* in the absence of HCs, and that CHC profiles across different beewolf species have similar compositions, indicating a widespread HC-based protection of defensive symbionts across Philanthini. While our findings support the important function of HCs in the AGS, we argue that AGS-mediated symbiont protection does not exert a conflicting selection pressure on the multifunctional HC profile and thus does not constrain its composition.

## 2. Results and discussion

**Figure 1.**
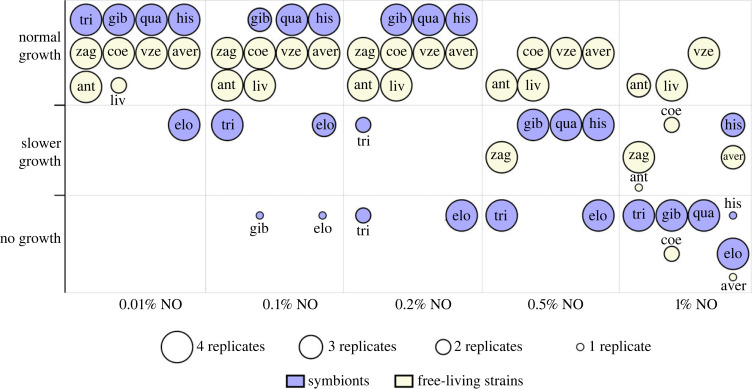
Nitric oxide (NO) sensitivity of ‘*S. philanthi*’ biovariations and free-living *Streptomyces* at different NO concentrations. Symbionts (purple) were more sensitive to NO than free-living *Streptomyces* (off-white) (ANOVA, *χ*^2^ = 103.5, *d.f.* = 2, *p* < 2.2 × 10^−16^), and growth inhibition increased with NO concentration (ANOVA, *χ*^2^ = 200.3, *d.f.* = 2, *p* < 2.2 × 10^−16^). In addition, the strains varied in their NO sensitivity (ANOVA, *χ*^2^ = 155.4, *d.f.* = 40, *p* = 1.618 × 10^−15^). The sizes of the circles indicate the number of replicates in the different growth categories. See electronic supplementary material, table S2 for strain designations.

We assessed NO sensitivity of five ‘*S. philanthi*’ biovariations from all host genera [21] and six free-living *Streptomyces* species (electronic supplementary material, table S2) to five NO concentrations. Survival was assessed as a trinary response (no growth, growing slower than control (without NO), growing as quickly as control). Most of the symbiont strains were already affected by low NO concentrations, with growth completely ceasing at 1% NO. By contrast, most free-living strains were unaffected at concentrations below 1% and still grew at 1% NO. In the statistical analysis, our final model retained ‘bacterial category/strain’ and ‘NO concentration’ as independent variables (electronic supplementary material, methods). Symbionts were significantly more sensitive to NO than free-living *Streptomyces* (ANOVA factor ‘bacterial category’, *χ*^2^ = 103.5, *d.f.* = 2, *p* < 2.2 × 10^−16^, figure 1), and strains varied in their NO sensitivity (ANOVA factor ‘bacterial category/strain’, *χ*^2^ = 155.4, *d.f.* = 40, *p* = 1.618 × 10^−15^). Expectedly, the impact of NO on bacterial growth increased with concentration (ANOVA factor ‘NO concentration’, *χ*^2^ = 200.3, *d.f.* = 2, *p* < 2.2 × 10^−16^). Given the symbionts' sensitivity towards NO, we hypothesize that other beewolf hosts beyond *P. triangulum* may use their HCs in the AGS to protect their symbionts from NO fumigation.

**Figure 2.**
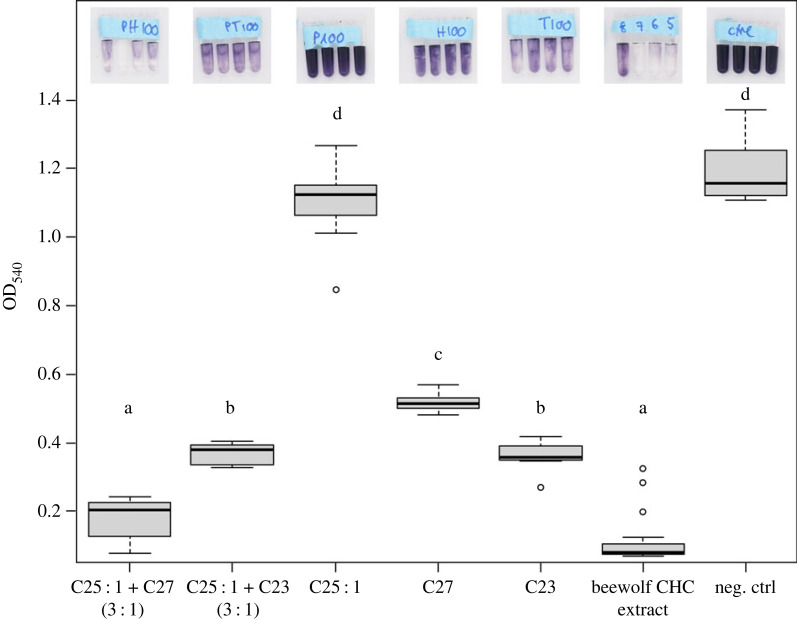
Reconstitution of the nitric oxide (NO) barrier effect with single and binary combinations of synthetic hydrocarbons (HCs). A total of 100 µg of HCs was applied for all treatments. Effectivity of HCs in blocking NO was measured as the change in coloration in an NO indicator solution, with higher OD_540_ values indicating stronger oxidation and thus less protection against NO by the HC layer (see representative images on the top; for all images see electronic supplementary material, figure S4). Beewolf CHC extracts served as positive, and hexane as negative control. All HCs and the alkene–alkane ratio of binary combinations are found in *P. triangulum* AGS and HC extracts (electronic supplementary material, table S1, figure S1). Letters indicate significant differences (Tukey's HSD, *n* = 8, *p* < 0.05).

To examine the link between HC composition and NO barrier function, we tested synthetic binary alkene–alkane (3 : 1) mixtures mimicking the AGS composition of *P. triangulum*, and their individual constituents for their ability to protect an NO indicator solution against oxidation. CHC extracts of *P. triangulum* (56–349 µg CHCs, x̅ = 161.0 µg) served as positive controls. Their alkene–alkane ratio diverged slightly from previous reports (electronic supplementary material, table S1, figure S1). Expectedly, beewolf extracts protected the indicator solution from oxidation. The degree of protection was not correlated with CHC amounts (Spearman's rank correlation, *n* = 19, rho = −0.335, S = 1522, *p* = 0.161; electronic supplementary material, figure S2), as they probably fell into the maximal range of protection. The combination of (Z)-9-pentacosene (C25 : 1) and heptacosane (C27) was as effective as the extracts (Tukey's HSD, *n* = 8, *p* < 0.05, figure 2), while C27, C23, and C25 : 1 + C23 (Tukey's HSD, *n* = 8, *p* < 0.05, figure 2) exhibited attenuated protection. The effect was still observed after a 10-fold reduction of the applied amounts of HCs (electronic supplementary material, figure S3). C25 : 1 did not prevent NO from reacting with the indicator solution (Tukey's HSD, *n* = 8, *p* < 0.05, figure 2).

Our experiments revealed that the hydrophobic NO barrier can be reconstituted by single and binary HC combinations and is not specific to certain HCs, implying a general effect. As previously observed in phase behaviour [22], binary combinations did not behave as predicted from individual HCs: While ineffective individually, C25 : 1 enhanced the effect of C27, but not of C23. C25 : 1 did not provide a barrier to NO, but C23 : 1-covered ‘*S. philanthi*’ survived an otherwise lethal NO exposure [8]. This may be explained by the difference in the HC amount applied in both experiments resulting from the need to apply the HC without a harmful solvent in the previous study. Interestingly, C25 : 1 + C27 protected better from NO than C25 : 1 + C23, although the latter more closely resembles *P. triangulum* CHC extracts [17].

Assuming a common site of production and/or a shared pathway for HC biosynthesis for the AGS, PPG, and cuticle, the HC composition is likely shaped by selection acting simultaneously on symbiont protection (AGS), prey preservation (PPG), and desiccation resistance (cuticle). The more complex profile of the AGS (as well as the cuticle and the PPG)—as opposed to a simpler mixture of C25 : 1 and C27—might be explained by the wider melting range of a more complex composition ensuring adequate viscosity or establishing a biphasic secretion under varying environmental conditions [23]. Furthermore, less abundant HCs may serve a role in AGS localization by the larva or as nutrients for ‘*S. philanthi*’ [17,24–26]. Alternatively, unspecific enzymes in the HC biosynthesis may produce a homologous HC series as a byproduct, without the minor HCs being selectively favoured [3].

In addition to *P. triangulum*, NO defence has been observed for the North American *P. gibbosus* and *P. basilaris* (M Kaltenpoth & T Engl, personal observation), suggesting a widespread distribution across Philanthini. Therefore, we assessed new and previously published HC profiles from available host species to speculate on their potential for symbiont protection from NO. CHC extracts of *P. histrio* were quantified using gas chromatography–mass spectrometry (GC-MS) and consisted of 76% alkenes/alkadienes, and 24% alkanes. Tritriacontene (C33 : 1), hentriacontene (C31 : 1) and hentriacontadiene (C31 : 2) accounted for approximately 67% of CHCs. Heptacosane (C27) and pentacosane (C25) (approx. 9% each) were the most abundant alkanes (electronic supplementary material, table S3, figure S5). Chain lengths of the dominant HCs varied across species (electronic supplementary material, table S3, figure S5). The alkene/alkadiene–alkane ratio of *P. histrio* resembled previously published profiles of *Trachypus elongatus* (64%/36%; electronic supplementary material, table S3, figure S5) [27]. Assuming that alkenes and alkadienes have similar physicochemical properties, it is also comparable to previously published alkene/alkane ratios of *P. triangulum* (C25 chemotype: 76%/23%; C27 chemotype: 72%/26%) [17] and *P. gibbosus* (87%/13%) [28].

Their equally alkene- and alkene/alkadiene-rich HC profiles may qualify other beewolves to provide a hydrophobic NO barrier to protect their symbionts if they fumigate their brood cells. The reported NO fumigation in *P. triangulum*, *P. gibbosus* and *P. basilaris*, and the ecology shared among beewolves renders a widespread NO fumigation across Philanthini wasps plausible.

Apart from blocking NO, similar HC profiles mediate prey preservation [5,6,12] and protect *P. triangulum* from desiccation. Previous studies indicate a selection for alkene-rich profiles in Philanthinae digger wasps, including beewolves (Philanthini) and part of the Cercerini, to efficiently preserve decay-prone Hymenopteran prey (e.g. Apidae and Halictidae) [29]. By contrast, basal Cercerini providing unembalmed Coleoptera possess diversifying HC profiles, with often lower amounts of alkenes [29]. Furthermore, Aphilanthopini are unlikely to embalm their ant prey, which is presumably less susceptible to microbial threats [28]. Thus, prey preservation likely evolved independently in the derived Hymenoptera–hunting Cercerini and in the ancestor of the Philanthini, the latter probably coinciding with the acquisition of defensive symbionts about 68 mya [30]. Although additional evidence is needed, we speculate that the evolutionary origin of NO fumigation and the HC-mediated protection of the symbionts may have coincided with the origins of symbiosis and prey embalming (electronic supplementary material, figure S6).

Prey preservation likely selects for high proportions of alkenes [29], and our experiments indicate their suitability for NO protection. For desiccation protection, the large qualitative variety of CHC in insects [3] suggests that this function can be realized by very different compositions, provided they form a biphasic layer [23]. Thus, HC-mediated protection of the symbionts does not appear to impose a conflicting selection pressure on the composition of the beewolf AGS that could otherwise compromise the efficiency of the same HC profile for desiccation resistance and prey preservation.

## 3. Methods

### (a) Bacterial cultivation

Bacterial strains were cultured in a 1 : 1 mixture of Sf-900 II SFM medium (Gibco, Thermo Fisher Scientific, Germany) and Grace's insect medium (electronic supplementary material, methods; Sigma-Aldrich, Germany) at 30°C in 24-well plates.

### (b) Comparative cultivation assay

We assessed the NO sensitivity of five symbiont strains representing all three host genera and different geographic origins [21], and six free-living *Streptomyces* strains obtained from DSMZ (Braunschweig, Germany). After NO exposure (electronic supplementary material, methods), we analysed the trinary growth observation (no growth, growth observed later than in the control, growth observed at the same time as in the control; *n* = 2–4) using a multinominal regression model. Growth was examined as a function of NO concentration and bacterial category (symbiont versus free-living *Streptomyces*), with ‘strain’ as a nested factor within bacterial category. Starting from the full factorial model, we used a step-wise reduction of model complexity to select the best-fitting model. Statistical analyses were performed in R i386 4.1.2 using the ‘nnet’ [31] and ‘car’ [32] packages.

### (c) Extraction and quantification of beewolf cuticular hydrocarbons

*P. triangulum* females collected in Berlin, Germany, were reared in observation cages [33]. We assessed CHC extracts from 19 females regarding their efficacy as an NO barrier. The females' antennae were removed. One female per extract was submerged in 1 ml hexane. After a 10 min extraction under stirring at RT, the female was removed, and hexane was evaporated under argon flow. CHCs were re-dissolved in 100 µl hexane. A 95 µl aliquot of each extract was evaporated under argon flow and stored at −20°C. The remaining 5 µl were used for GC-MS (electronic supplementary material, methods). We characterized the CHC composition of *P. histrio* using a single female from a collection near Knysna, Western Cape Province, South Africa, in 2005. After removing the head, the thorax and abdomen were extracted for 30 min in hexane. The extract was subjected to GC-MS (electronic supplementary material, methods).

### (d) Cuticular hydrocarbon experiments

We purchased HCs from Sigma Aldrich, Germany, and Cayman Chemical, Michigan, USA. C25 : 1 was combined with C23 or C27 in a 3 : 1 ratio to mimic the alkene–alkane ratio found in beewolves. We transferred 10 µl of hexane containing 100 µg, 50 µg or 10 µg of each treatment (*N* = 7–8 each), on top of 40 µl NO indicator solution (electronic supplementary material, methods) in tubes (diameter = 3 mm; Biozym, Germany), respectively. As a positive control, we transferred each beewolf CHC extract in the same way. Indicator solutions treated with 10 µl hexane served as negative controls (*N* = 8). The applied HCs form a distinct layer on top of the indicator solution due to their hydrophobicity. After NO exposure (electronic supplementary material, methods), the content of the tubes was centrifuged in 0.5 ml tubes at maximum speed for 30 s. We measured the OD_540_ of 20 µl of the supernatant in a 384 well plate (VarioSkan Lux, Thermo Scientific, Germany). We performed OD_540_ comparisons across all 100 µg treatments and both controls, and within treatments, using a one-way ANOVA. The correlation between the amount of beewolf CHCs and the OD_540_ was analysed using Spearman's rank correlation. Statistical analyses were conducted in R (V4.15).

## Data Availability

The raw data underlying this publication are available from the Edmond repository of the Max Planck Society: https://doi.org/10.17617/3.HPDVQM [34]. Supplementary material is available online [35].
